# A Method for Assessing the Reliability of the Pepper Robot in Handling Office Documents: A Case Study

**DOI:** 10.3390/biomimetics9090558

**Published:** 2024-09-16

**Authors:** Marius Misaroș, Ovidiu Petru Stan, Szilárd Enyedi, Anca Stan, Ionuț Donca, Liviu Cristian Miclea

**Affiliations:** 1 Faculty of Automation and Computer Science, Technical University of Cluj-Napoca, 400114 Cluj-Napoca, Romania; marius.misaros@aut.utcluj.ro (M.M.); ovidiu.stan@aut.utcluj.ro (O.P.S.); ionut.donca@aut.utcluj.ro (I.D.); liviu.miclea@aut.utcluj.ro (L.C.M.); 2 Faculty of Industrial Engineering, Robotics and Production Management, Technical University of Cluj-Napoca, 400114 Cluj-Napoca, Romania; anca.stan@muri.utcluj.ro

**Keywords:** AI, communication, humanoid robot, interaction, MTBF, pepper robot, reliability, service robot

## Abstract

Humanoid robots are increasingly being utilized in various activities involving humans, as they can facilitate certain tasks and provide benefits to users. Humanoid service robots possess capabilities akin to human performance, often proving advantageous due to their operational speed and immunity to fatigue. Within the scope of this study, Pepper, a humanoid robot renowned for its fidelity in mimicking human gestures and behavior, serves as the focal point. Tasked with aiding office occupants in object manipulation and relocation, Pepper underwent a targeted reliability assessment. This assessment encompassed the development of a reliability block diagram (RBD), alongside meticulous analyses of individual components and system functionality across diverse operational scenarios.

## 1. Introduction

The use of robots has become ubiquitous in modern society, serving as indispensable tools that enhance the quality of life and optimize daily activity. As technology has progressed, there is a growing demand to simplify routine tasks, coupled with the human inclination for continuous learning. As a result, the integration of robots into various sectors of society has seen a surge, particularly in roles involving interaction with humans, notably within the service and industrial sectors [1], automatically increasing the level of sustainability for technology companies [2].

The concept of human–robot cooperation serves as the fundamental premise from which this research initiative originated. Moreover, it is a concept that is gaining increasing attention and practical application in more and more areas of activity of contemporary society. The use of assistive robots in both domestic and professional settings has the potential to significantly enhance productivity, thereby extending the duration of independent living for elderly persons, who may otherwise require human assistance [3].

Soon, robots will become more prevalent in people’s daily lives. The trend towards advanced home robots with new functionalities is becoming more evident, even within domestic settings. These robots will act as roommates, offering assistance whenever needed, and may even serve as companions for elderly individuals requiring consistent and reliable support. Their integration into work environments can be achieved without significant structural modifications, as they possess the capability to dynamically interact and perform physical tasks like those undertaken by humans [4].

While the field of robotics encompasses a wide array of applications, studies indicate that older age segments are less receptive toward incorporating robots into the caregiving process compared to younger segments. However, they remain open to the idea of introducing them into their lives to make things easier. Substantial progress has been made in recent decades to foster improved interaction partnerships, integrating new behavioral patterns, and thus achieving a deeper human–robot connection [5], which has also qualitatively influenced economic development [6]. In order to enhance the reliability of the robots’ systems for perceiving the environment in all activity postures, including turning, bending, and manipulation, a study was conducted with the objective of eliminating image jerk in these motion situations. This was achieved through a method that focuses on the points of interest within the image [7]. A study was conducted to evaluate the reliability of the robots in question. The analysis of thirteen robots yielded several noteworthy results. Notably, the robots exhibited a high degree of unavailability, with an average mean time between failures (MTBF) of only eight hours [8].

The present research appoints a Pepper robot, specifically designed for human interaction, to ascertain the reliability of the robot in performing tasks that an employee typically reproduces in a similar and repetitive manner. Additionally, the research aims to achieve several secondary objectives, including the facilitation and optimization of work processes, the enhancement of communication at colleague level, and the promotion of positive effect in monotonous activities. From the perspective of potential discrepancies that may arise during the robot’s operations, these can be classified into two primary categories: those originating from the interactions of individuals and those that are external stimuli. In terms of obstacles to interacting with employees, communication errors with the robot or evasive actions that could result in task failure may be identified. External stimuli that impede the robot’s performance may manifest in various forms. For instance, obstacles that hinder the robot’s movement or the presence of certain auditory stimuli, such as loud noises or voices, can disrupt the robot’s operation. Similarly, light sources that are not clearly defined may also interfere with the robot’s perception of its surroundings.

## 2. Service Robots Literature Review

Gonzalez et al. [9] presume that service robots are categorized according to the field in which they operate, encompassing a subset of advanced robotics. The International Federation of Robotics (IFR) emphasizes the importance of robots belonging to this field to benefit from semi- or fully autonomous behavior [10]. A more refined definition is provided by the International Organization for Standardization (ISO), which, through its committee of experts in 2007, highlighted their use as a utilitarian entity that performs precise tasks for humans or equipment, explicitly excluding applications used in industrial automation [11]. The ISO 8273 standard further elucidates the difference between industrial robots and service robots [12].

According to these standards, service robots are classified into two main categories. The first one includes robots designed for personal or domestic purposes, including caregiving for the elderly or disabled, performing normal household tasks, surveillance, transportation, and other tasks within similar contexts. The second category comprises robots intended for professional use distinguished by their engagement in activities pertinent to professional domains (professional cleaning, construction, medical robotics, maintenance and inspection systems, underwater systems, security and defense systems, and others). Notably, these robots operate autonomously and do not require a specialized operator, unlike conventional robots [13]. A study based on the users’ perspective on the activities performed by robots in the domestic domain revealed that the manipulation of objects is an essential skill for humanoid robots to provide a real service. This finding naturally leads to the conclusion that this type of robot can provide social-friendly services [14].

A major factor driving the substantial evolution of service robots is directly linked to the fourth industrial revolution. There is currently a wealth of information that can be accessed at the click of a mouse by accessing various libraries. Also, a substantial factor is the speed at which technology is evolving, which has a significant impact on the many systems that have adopted new concepts, fostering paradigm shifts from a narrower concept to a more developed and efficient one [15], thus favoring research and innovation for a transition to sustainability [16]. Incorporating artificial intelligence (AI) into service robots promises significant cost savings and increased productivity, thereby encouraging the increased adoption of such robots as well as an increased interest in robotics research. Service robots are a new area of research because of their disruptive and distinctive characteristics. In order to facilitate a more comprehensive understanding of the various categories of service robots, it is instructive to consider the classification system proposed by the International Federation of Robotics. This system distinguishes between two principal categories of service robots: personal service robots and professional service robots [17]. In their article, Papageorgiou presents a personal robot service application designed to assist elderly or sick individuals [18]. The proposed system employs a humanoid robot that is continuously available to provide support, highlighting the potential benefits of such a robot in addressing the needs of vulnerable populations. The primary focus of the case study conducted by the researchers is to aid individuals with locomotor disabilities. The advent of personal service robots has also permeated the domain of domestic activities, as evidenced by applications such as cleaning, as illustrated in [19]. These robots provide users with a hygienic and well-maintained living environment, thereby enhancing their quality of life. Additionally, they are utilized for recreational purposes, as exemplified by Cosentino in [20], where a case study demonstrates that the robot assesses the user’s emotional state and responds accordingly, facilitating a tailored interaction. From the perspective of professional service robots, they are utilized in a multitude of applications, including the facilitation of shopping experiences. In such settings, these robots are employed to assist customers in navigating the selection process and identifying optimal products [21]. In [22], Psarros outlines the advantages of these robots in underwater applications, which provide a safer alternative for humans and facilitate access to otherwise inaccessible locations. Professional robots are also utilized in restaurant settings to provide customer service, as evidenced by the application developed by Qing-xiao in [23]. The case study presented in this manuscript is categorized as a professional service robot, as defined by its ability to operate in a work environment with a non-restrictive number of humans in the operation.

As to whether service robots can provide the same quality of service as human staff, a study has been carried out to assess their reliability and responsiveness, which clearly shows that the reliability of a service robot is higher than that of human staff [24]. The utilization of service robots in the tourism industry is becoming increasingly prevalent, with the objective of deepening their involvement in this sector. Lu Lu, in [25], conducted a research analysis on reliability, which yielded findings that corroborate the dependability of these robots. The reliability of the service robot in this domain exhibited results above the lower limit of 70%, with precise outcomes ranging between 89% and 96%.

In [26], the authors address a critical topic in robotics: the assessment of robot quality by adding mechanisms designed to contribute to the improvement of the whole system. During the pandemic, Lin’s team developed a service robot [27] that optimized waiting times for serving food and maintaining hygiene. In [5], the current trend of service robots is presented to provide a comprehensive perspective. Table 1 summarizes the main trends.

## 3. Interactive Humanoid Robots

### 3.1. Interaction between Robots and Humans

Robots offer a straightforward solution to assist with various activities, including providing support to individuals in their later years. Beyond their utility, they offer the added benefit of availability, capable of executing a spectrum of tasks in daily life, no matter the time of the day [28].

Research has shown that robots with a human-like or small animal-like appearance are more effective in interacting with users. However, while appearance plays a role, the characteristics and capabilities of the robot exert a greater influence on users. Notably, predictions regarding robot appearance vary among individuals, often influenced by their personality traits. As humanoid robots have become more adopted into society, they have acquired the ability to exhibit body language and other social cues characteristic to humans. This allows robots to perform actions that were previously only possible for humans [29].

Ya-Huei et al. contend that robots designed for specific tasks should not strive to accomplish goals autonomously. Rather, they should assist humanity in developing their skills. This would increase efficiency and productivity, freeing people from dangerous or repetitive tasks [30].

Successful interaction between a robot and a human requires a substantial degree of trust on the part of the user. Therefore, the users must perceive that their robot counterpart has their best interests at heart and that collaborative actions will ultimately prove beneficial. This need for trust becomes particularly evident in high-risk missions, where users entrust their safety to the robot’s suggestions, which, in some cases, may exhibit greater accuracy than human perception [31]. The level of trust in some interactional situations can serve as a strong advantage. The result of a very good cooperation can be seen in the testimonies of military personnel engaged in explosive ordnance disposal, who laud the performance and cooperation of robots to the extent of bestowing them with names (e.g., Sgt. Talon) and even Purple Hearts commendations for exceptional service [32]. In most cases, the studies focus on a more precise instruction leading to a single human–robot interaction [33].

A humanoid interaction robot must be designed in such a way that it is always able to perform the necessary tasks successfully. This is encapsulated in its reliability requirements. In general, there is a certain degree of trade-off between reliability, maintainability, and safety in an interaction. The safest robot is therefore the one that does nothing. In certain instances, the availability and reliability of the robot may be superseded by the necessity to prioritize human welfare. This is particularly pertinent in the context of robot utilization in applications such as surgery or rescue operations [34]. One aspect that is advantageous for the method of cooperation in services is the benefit of being able to achieve a certain personalization of the interaction, which would give the customer an advantage, so that they perceive it as receiving a special service. When used in social services, the advantage of personalization increases the level of cooperation, interaction, and general acceptance [35].

### 3.2. Core Concepts of the Pepper Robot

Pepper the robot has a friendly and approachable appearance, inspiring confidence and a sense of safety. Its design features googly eyes, a rounded childlike head, and small arms, creating a human-like impression while still maintaining its unique robotic identity [36].

Pepper is designed to have a childlike appearance, standing at only 120 cm tall, which is typical of a six-year-old. This allows it to easily integrate into its surroundings and interact with users in a way that is psychologically pleasing. Its natural arm movements and gestures further enhance its ability to interact confidently with users. From the outset, Pepper’s developers aimed to create a harmless, friendly, and controllable robot that would encourage playful and creative interaction with many people [37].

Although Pepper’s appearance bears human resemblance, its locomotion relies on three wheels instead of bipedal walking. Additionally, Pepper is equipped with a tactile screen on its chest, two conventional cameras, and four microphones in the chest area to intercept sounds. The device is equipped with two ultrasonic sensors, six laser sensors, and three shock sensors, all mounted on its wheels, allowing it to detect and navigate around potential obstacles with ease [38].

The Pepper robot has 20 degrees of freedom, allowing for movement and high flexibility. It has a total of 17 joints and navigates on three wheels. The degrees of freedom are distributed as follows: one in the head, one in each shoulder and elbow, one in each hand and five in the fingers, two at the hips, one at the knee, and three at the base. The robot’s wheels are omnidirectional, enabling it to move even when climbing a 1.5 cm step or a slope of up to 5 degrees. Softbank designed the actuators using DC motors for the upper limbs and a brushless DC motor for the lower limb [39].

The robot’s body parts are interconnected between a fixed and a moving part, with the torso serving as the stationary component and the outermost part being movable. The head joint has a range of movement from −119.5 to 119.5 degrees around the *z*-axis and −40.5 to 36.5 degrees around the *y*-axis.

To manipulate objects, the robot arm articulation follows a similar principle to the main articulation of the head. The relevant parts of the torso remain fixed, while the outermost parts are movable. The left shoulder joint has a movement range of 0.5° to 89.5° relative to the *y*-axis, and a twist range of −119.5° to 119.5° relative to the X’ axis. The hand wrist joint has a range of −104.5° to 104.5°, and the left elbow joint has a range of −89.5° to −0.5° in relation to the Z’ axis.

The robot was introduced to society in the summer of 2014 and has since made considerable progress. Its development relied on the Python programming language, enabling the seamless control of its sensors and software connections [40].

The deployment of the Pepper robot in the office setting is explored in [41] with a particular focus on the necessity for inter-colleague collaboration during the pandemic. This research resulted in the development of a series of interactive services that required differentiation between users. Through its ability to recognize, speak, listen, and move, the robot enabled natural cooperation with people, leading to efficient task management. To evaluate the implemented system, a video survey was conducted, in which the robot provided assistance in an office on the campus of a university. The results indicated that 92% of participants evaluated the robot positively. In [42], Okafuji integrates the robot into the office environment in a manner that maintains the user’s attention. This was achieved by the robot greeting colleagues in a socially acceptable manner and maintaining eye contact, which is an automatic indication of social acceptance. Over the course of a week, the approach has been progressively more successful, with an increasing number of promising interactions. Additionally, the robots were employed in the training of new employees. A study was conducted in collaboration with a company with the objective of implementing two distinct training methodologies: one conducted by the robot and the other presented on a digital display. Those who underwent training with the assistance of the robot reported an enhanced level of satisfaction and attention during the presentation. Half of the respondents indicated a preference for robot-based training in the future, while the other half expressed a preference for the more traditional, instructor-led approach [43].

In the case study on which the inter-system reliability analysis is carried out, the Pepper robot has to fulfill a series of conditions regarding its activity. Its task is to recognize the people who come to work and to receive voice commands from office colleagues so that, at the moment of reception, it will move to retrieve the necessary documents and store them in the designated place.

## 4. Materials and Methods

In the process of designing and developing a system, it is of the utmost importance to assess and mitigate the risk of failure in terms of the economic and social consequences that may arise. It is of the highest importance to predict the occurrence of a fault-causing component as accurately as possible by developing the most effective maintenance system; otherwise, the system may result in extremely dangerous disasters. Failures can result in the loss of functionality of a system, which can then lead to a cascading series of failures, ultimately leading to the degradation of the entire system developed. In some cases, this can result in injury to certain users. This entails a number of factors, both economic and legal, as well as factors related to trust, which is likely to decrease. This is accomplished through a comprehensive examination of the system’s reliability and security [44]. Reliability refers to the ability of an item to perform its intended function under given conditions over a predetermined timeframe. It can be analyzed qualitatively as the ability of the item to remain functional [45] and is computed using (1). In this context, λ represents the failure rate, while t denotes the time period over which the reliability is calculated.

(1) $$R(t)=e^{-\lambda t}$$

Reliability is an important factor in the development and design of a robot, which has attracted increased attention in terms of academic interest in this topic [46]. Several studies have been carried out to find the optimal approach for managing uncertainties associated with structural parameters and rotational joints [47].

**Figure 1:**
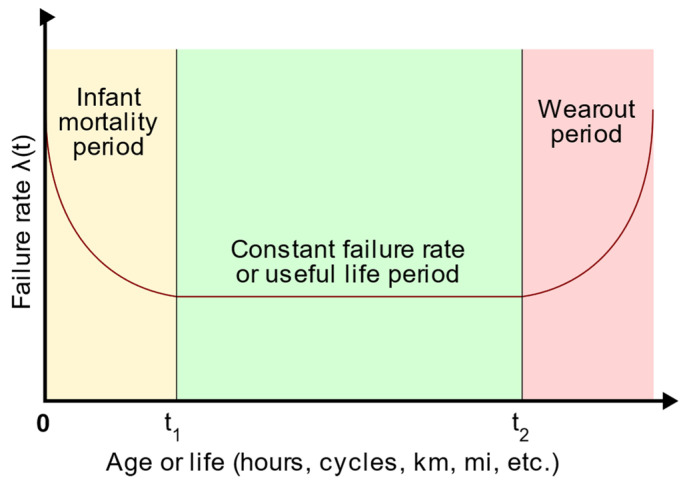
Idealized hazard rate bathtub curve.

The failure of a given set of products calculated as a function of r(t), post-sale, may be due to internal weaknesses in design, manufacturing, usability, or maintenance policies that have been violated. The hazard rate, denoted as λ(t), represents the number of unaffected products remaining at a given time, t. Figure 1 shows the bathtub curve, which is an idealized form of the hazard rate of a product.

The graph above has three distinct stages: the infant mortality period, the constant failure rate or useful life period, and the wear-out period. During the initial phase, the hazard rate declines and stabilizes at time t1, when the weak products have failed. The subsequent useful life period denotes a phase wherein the population of products reaches a consistent level, corresponding to the lowest hazard rate. Following this, time t2 indicates the end of the useful life and the beginning of product attrition. Notably, the failure rate exponentially increases during this final phase, signaling the necessity for product replacement [45]. To evaluate the performance of repairable faults, a prevalent metric of reliability is the mean time between failures (MTBF). To increase the MTBF, it is common practice to perform periodic system maintenance checks [48]. MTBF is a reliability metric estimating the duration a robot can operate without experiencing failure [8]. The MTBF metric is used to evaluate hardware components or systems. If it has a high MTBF, it indicates that there is less chance of failure and that it is a reliable system or piece of equipment. This index is used to determine the maintenance intervals of equipment, resulting in the preventive risk of failure [49].

(2) $$MTBF=\frac{Number\;of\;Hours\;the\;Robot\;was\;in\;Use}{Number\;of\;Failures}$$

MTBF can also be calculated as:

(3) $$MTBF=\frac{1}{\lambda }$$

Here, λ is the breakdown intensity. 

A graphical analysis technique is used to analyze the reliability of a system on a component-by-component basis by logically connecting all the components that make up the entire system [50]. This method, using a reliability block diagram (RBD), consists of all the necessary components of the system. RBD serves as a systemic representation illustrating the impact of component failures on overall system performance contingent upon the individual reliability of the system components, denoted by R_i_. The complexity of RBD diagrams is directly influenced by the number of components, m, and they are realized in two distinct forms:

- Series, which is represented by the Formula (4);

(4) $$R\left(t\right)=\prod_{i=1}^{m}R_{i}(t)$$

- Parallel, which is represented by the Formula (5) [8];

(5) $$R\left(t\right)=1-\prod_{i=1}^{m}\left(1-R_{i}(t)\right)$$

**Figure 2:**
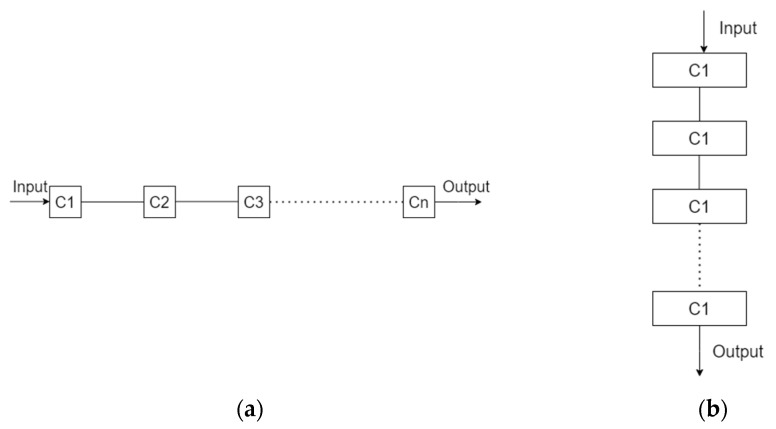
Component connection diagram (**a**) Series, (**b**) Parallel.

If the individual components within a system are connected in series (Figure 2a), the failure of any single component will result in the failure of the entire system. Conversely, if the components are connected in parallel (Figure 2b), the system will remain operational until all components have failed, with the failure of one component not impeding the functionality of the system [51].

In general, activities related to the reliability of a product can be divided into three main development phases:

- Concept phase;
- Design phase;
- Production phase.

A risk and safety analysis of the causes of the system is conducted through the utilization of a Fault Tree Analysis (FTA) diagram. The diagram contains all the system components that have the potential to result in an adverse event or system failure. This is achieved through the construction of a logic tree, which begins with an adverse event and progressively decomposes into causal events [52].

In order to ascertain the evolution of the system over time, a Markov chain analysis was conducted on the system. This analysis demonstrated that the probability of the system reaching a subsequent state is contingent upon its current state. Such calculations are utilized in reliability studies to determine the probability of a system being in a functional or faulty state. This, in turn, enables the estimation of the system’s lifetime [53].

This process is performed both in terms of the whole system and independently for each of the components [54]. In accordance with this model, an investigation was conducted into the reliability of the entire system, commencing at the conceptual level of the individual components integrated within the system. During the development of the diagrams, the design of the entire structure on the evaluation branches was carried out, followed by the actual calculation part in order to determine the final result.

## 5. Results

Deep neural networks have recently achieved significant success in speech and image recognition, thanks to the use of large amounts of training data [55]. The Pepper robot can interpret external stimuli and voice commands to perform specific actions. Pepper can process voice commands and translate them into a trajectory that leads to the completion of the assigned task. To orchestrate the entire action, a predefined set of steps is followed. These steps not only involve executing the interaction but also interpreting certain states autonomously. This interpretation helps in decision-making and carrying out the action correctly. This action is automatically influenced by external environmental factors [56].

To achieve these functionalities, the Pepper robot is programmed with a dedicated software called Choregraphe 2.8.7.4, which facilitates the development of various applications. Choregraphe is a versatile application that enables the implementation of different behaviors. These behaviors can be simulated on a virtual robot within the application or loaded onto a real robot, as demonstrated in this study. The entire task execution path is developed by using and creating new application-specific boxes using the Python programming language.

The NAOqi 2.8.x software development kit and documentation define the operating system integrated into the Pepper robot. They allow for the interpretation and implementation of structures developed in Choregraphe on the robot itself [57].

### 5.1. Formulating the Mathematical Components

The objective of the presented study is to enhance office productivity by optimizing document retrieval and forwarding for specific users. This will be achieved by developing and implementing new functionalities for the Pepper robot. To accomplish this task, a set of functionalities must be incorporated. The modules utilized for this purpose are as follows:

- Face recognition;
- Object recognition;
- Face Tracking;
- Timeline.

**Figure 3:**
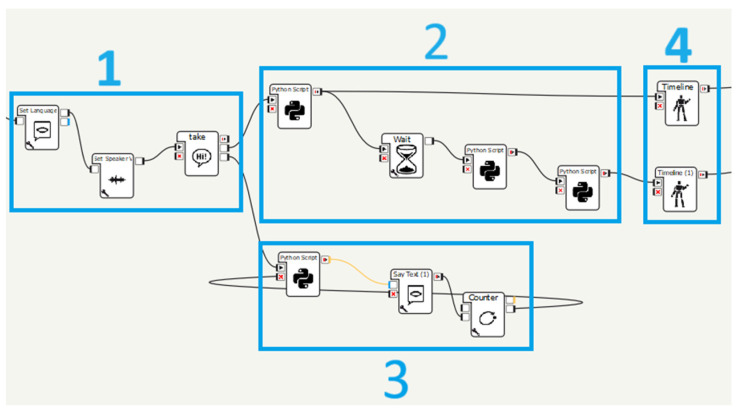
Application overview in Choregraphe.

The system is implemented using the configuration development method in the Choregraphe suite. The diagram aims to meet the requirements and objective functions of this research. It consists of interactive components that the robot can operate at user-defined times (Figure 3). The main diagram is divided into four stages, each playing a key role in the application flow:

- The dialogue that presents the first part of the scheme;
- Face and object recognition—third part of Figure 3;
- The movement that is represented as the second part in Figure 3;
- Manipulation, shown as the fourth part in Figure 3.

When the robot is ready to initiate section 1 of Figure 3, its eyes should illuminate in blue, signaling its readiness to receive a voice command from the user. Upon successfully capturing a voice command, the robot’s eye will transition to green, initiating the intended activity. If the command is not successfully intercepted, the robot’s eyes will turn and remain red until the command is correctly interpreted.

**Figure 4:**
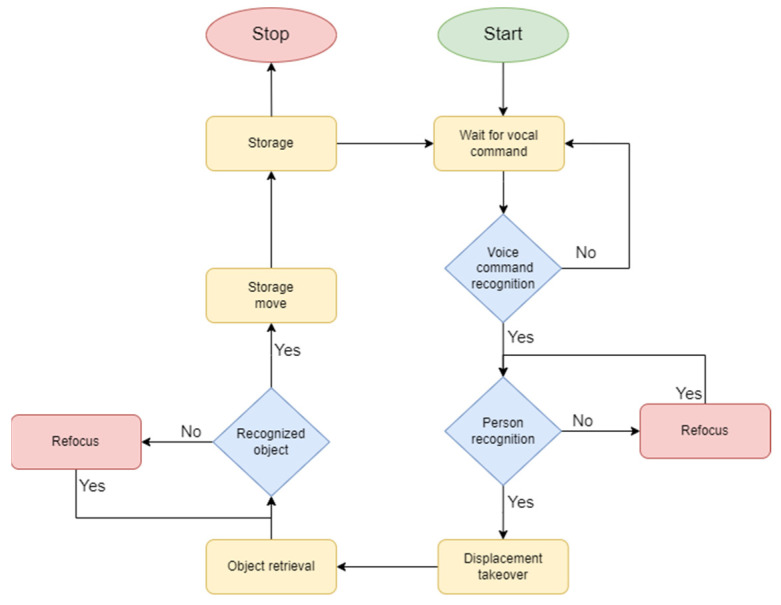
Flowchart of application process.

After confirming the interception of the voice command, the robot proceeds to execute the sequence of activities depicted in Figure 4.

**Figure 5:**
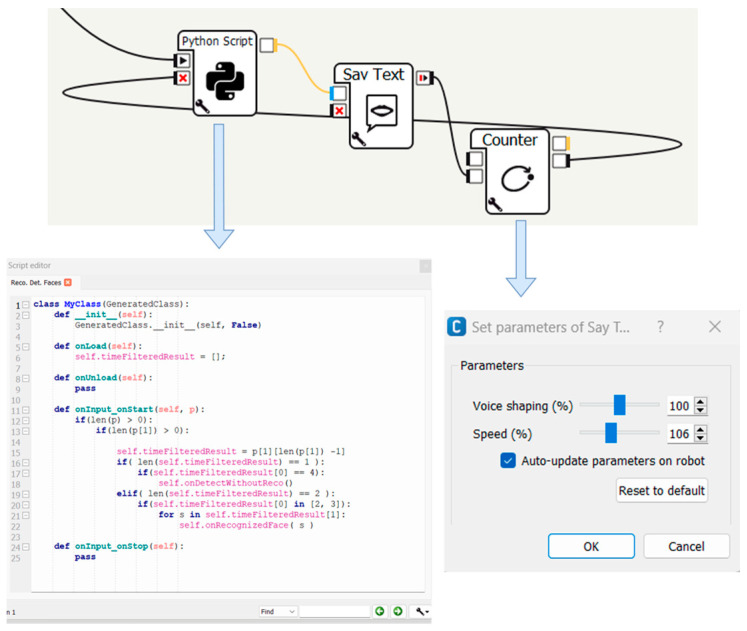
Recognition process.

In this scenario, the robot’s memory contains several potential users. Upon initialization, the Pepper robot will begin identifying the user who wishes to interact with it when the user says “hello”, as shown in Figure 5.

**Figure 6:**
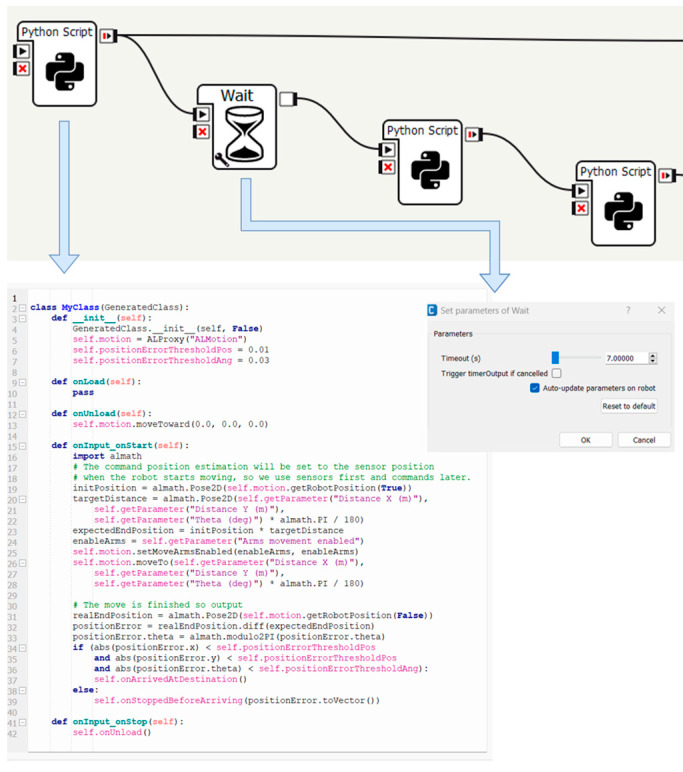
Robot movement and control.

Mobile robotics requires the skill of visual navigation to move through dynamic environments by avoiding obstacles and reaching the target [58]. The movement of the robot to retrieve the object aligns with step 2 in the diagram shown in Figure 6. Once the person who gave the command is recognized, the robot will move to pick up the object. The robot will position itself at an optimal distance from the user to allow for an unconstrained pick-up maneuver and the ability for the user to turn towards the next direction of movement after picking up the object.

**Figure 7:**
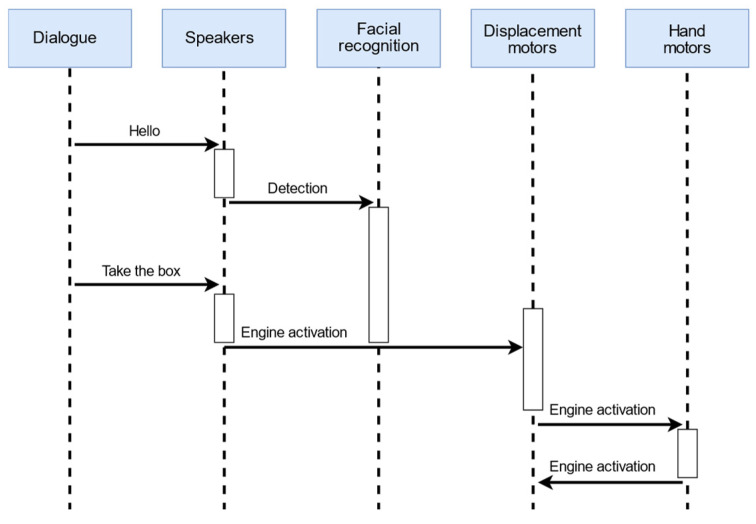
Process sequence diagram.

In accordance with the sequence diagram depicted in Figure 7, the robot follows a sequential pathway to execute the action, using a fluid and human-like movement. This benefits the user by making the action easier to understand and follow.

To ensure the system’s stability during development and testing, it was necessary to validate the camera visibility and robot trajectory. Upon confirming that the environment did not impede the robot’s functionality, it proceeded to “memorize” the faces of individuals in the office for interaction. Once this was accomplished, the experiment phase started by initiating the dialogue with Pepper. Following the sequence outlined in Figure 7, the robot will confirm the successful receipt of the voice command and then proceed with recognition and movement. The next step involves the robot reaching the user and positioning itself in a manner that allows the user to begin picking up and handling the object. The final step displays the sequence in which the Pepper robot navigates the last point of its mission to deposit the received object.

**Figure 8:**
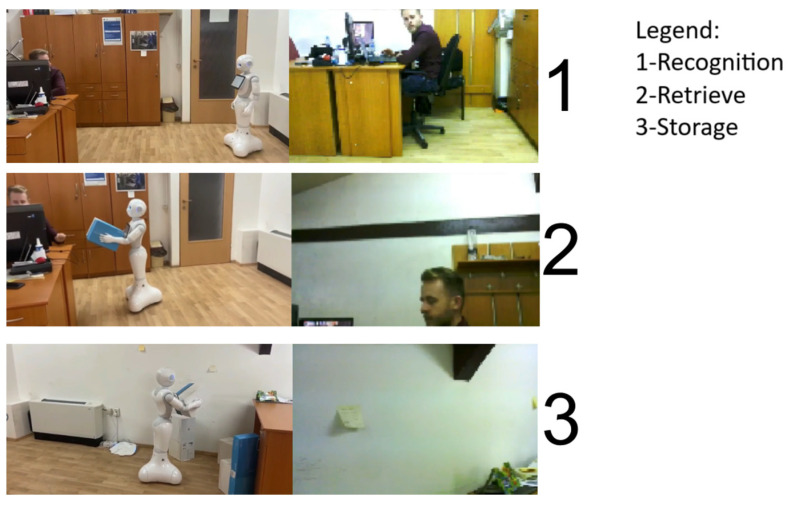
Interaction with the Pepper robot.

Figure 8 illustrates the three stages from both the robot’s perspective and the external environment’s.

The images on the right-hand side show the robot’s view of the environment through its forehead camera. On the left-hand side of the figure, the same action sequence is attached, showing the entire scene from the external environment.

The robot was used to simulate human interaction in an office environment with non-specialists. Despite the challenging conditions, the Pepper robot performed as it would in a normal environment, accounting for any external impediments that may arise during the task.

### 5.2. Assessment of Reliability

The PTC Windchill Quality Solution software 11.0 is used to model the reliability of the entire system. This software is known for its excellent processing capabilities when calculating system availability, maintainability, and reliability. It allows for analysis in various system settings, providing valuable insights into the system’s performance across different working conditions.

**Figure 9:**
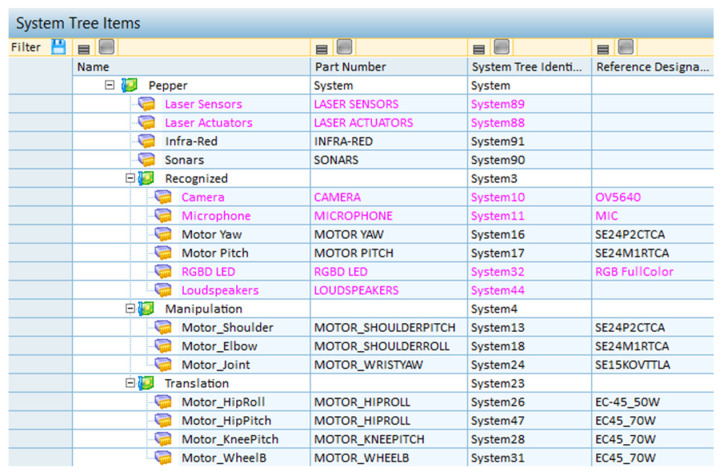
System tree architecture.

The system architecture was designed by assembling its components and subcomponents to perform the computation. In order to perform the requisite calculation, a time period of t = 10,000 h was established, and each component was incorporated based on its specific benefits. The architecture is divided into three main parts (Figure 9).

The first component recognizes the person giving the command and receives the voice command. The second component contains the motors responsible for moving the robot. The third component is responsible for the robot’s ability to grasp and manipulate objects, which is made possible by its motors.

**Figure 10:**
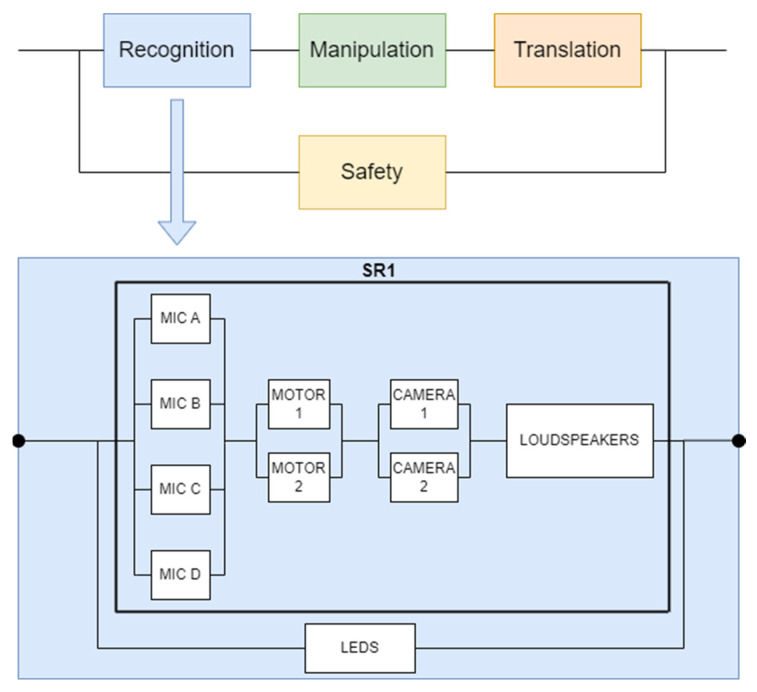
RBD diagram for recognition.

Figure 10 presents the interconnections among the subsystems composing the overall functionality of the robot, and the RBD for recognition in particular.

#### 5.2.1. RBD for Recognition

In order to ascertain the reliability of a given system, it is essential to analyze the MTBF figures provided by the manufacturer for each individual subcomponent within each subsystem. This can be achieved by utilizing the relevant formulas, specifically (1) and (3). If the probability of failure F(t) is complementary to R(t), we have:

(6) $$R\left(t\right)=1-\left(1-SR1\right)\left(1-LED\right)$$

Figure 10 shows the RBD diagram, which highlights the mode of operation and the series/parallel connections of each component module of the system. This diagram is based on the operation or fault condition of the component to evaluate the reliability. The main RBD diagram consists of four modules as follows: detection, manipulation, translation, and tracking. The first three modules are serialized, which means that if one of them fails, the process cannot be completed. The fourth component is called security; it is responsible for the security modules that are present throughout the system. The action can be realized even if this security component is not functional, but only if the system is ideal and does not encounter any disturbing factor in any of the three execution phases. The RBD diagram can be used to determine whether new modules need to be added at a later stage, and a reliability calculation based on the added components can be used to determine whether the added modules have an operational advantage or disadvantage. One of the main advantages of using this diagram is the ease of extracting or adding a new component to the system, which can be achieved by simply restructuring the diagram. Another advantage is the impact analysis of defects that may appear. In the case of large systems, these diagrams present a disadvantage in terms of high complexity; at the same time, for some systems, these diagrams represent an oversimplification.

In order to calculate the reliability of each module shown in the RBD, an independent diagram was made for each section so that there is the possibility of independent calculations on each of the four sections.

In order to ascertain the failure rate, which is a representation of the frequency of occurrence of faults in a system comprising a recognition component, a series of formulas were employed, (1), (3), (4), and (5), which resulted in Formula (7):

(7) $$\lambda =-\frac{\ln\left[1-\left(1-e^{-\lambda_{sr1}t}\right)\left(1-e^{-\lambda_{led}t}\right)\right]}{t}$$

#### 5.2.2. RBD for Manipulation

**Figure 11:**
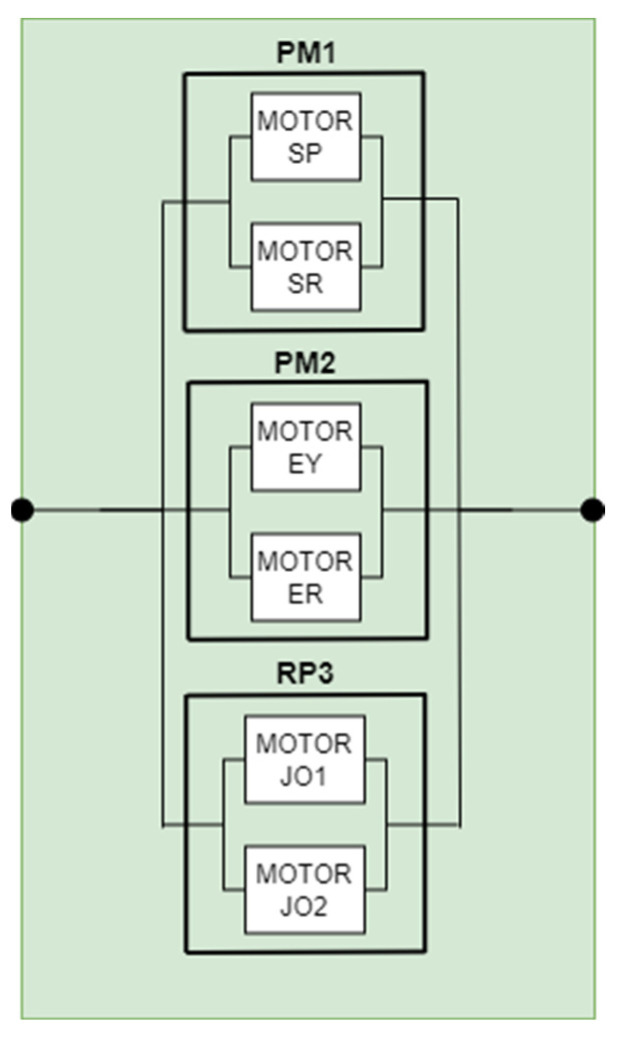
RBD manipulation diagram.

The second component, which forms part of the principal RBD diagram of the entire system, is responsible for manipulation (see Figure 11). The apparatus comprises six motors, which serve three junctions of the two robot arms. The first category of components is responsible for the shoulder joints, where the two “ShoulderPitch” and “ShoulderRoll” motors are utilized. The second category consists of the “ElbowYaw” and “ElbowRoll” motors, which are responsible for the joint maneuvering at the elbow of the hand. The final category of components is responsible for maneuvering the palm and fingers, which is accomplished by using the “WristYaw” and “Hand” motors.

The reliability of the manipulation subsystem is calculated in accordance with Formulas (1) and (3) utilizing the values of the user-defined components, as shown in Formula (8):

(8) $$R\left(t\right)=1-\left(1-PM1\right)\left(1-PM2\right)\left(1-PM3\right)$$

In order to calculate the failure intensity of the manipulation subsystem, the equations and Formulas (1), (3), (4), and (5) were employed, resulting in Formula (9).

(9) $$\lambda_{manipulation}=-\frac{\ln\left[1-\left(1-e^{-\lambda_{pm1}t}\right)\left(1-e^{-\lambda_{pm2}t}\right)\left(1-e^{-\lambda_{pm3}t}\right)\right]}{t}$$

#### 5.2.3. RBD for Translation

**Figure 12:**
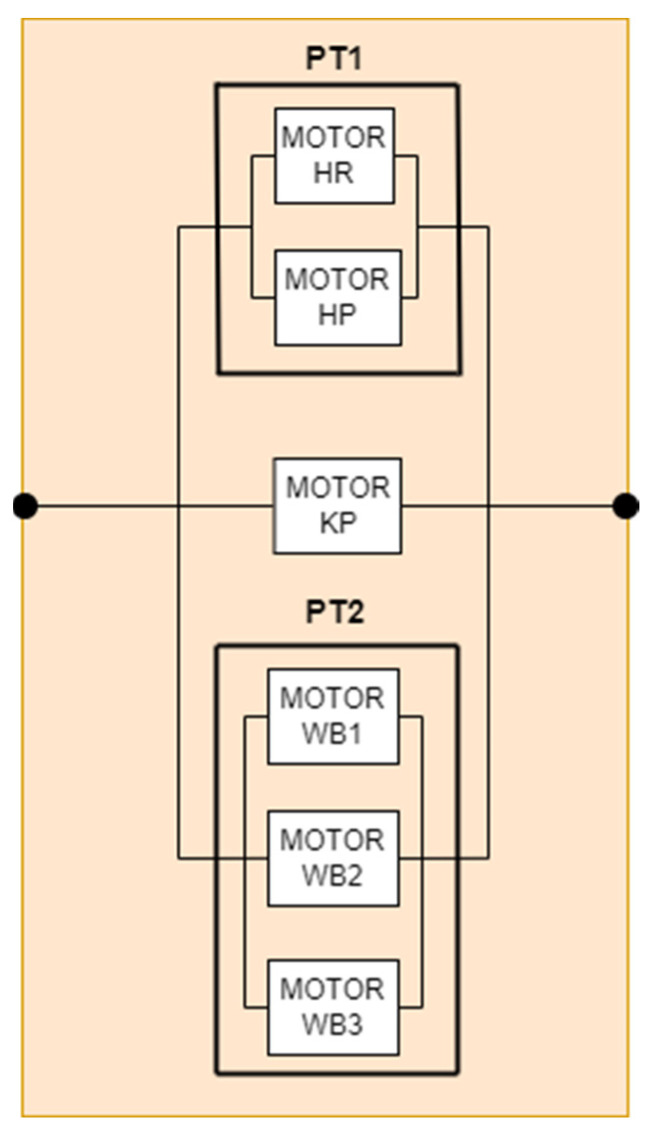
RBD diagram for translation.

The final component of the system is designated “Translation” and is tasked with facilitating the robot’s navigation between the designated destinations (see Figure 12). The system comprises six motors, each of which ensures the linear and uniform translation of the robot. The components that comprise this subsystem can be classified into three categories. The first category comprises the “HipRoll”- and “HipPitch”-type motors, which are responsible for the movement of the robot’s hips in the vertical plane. The second category is composed of a single “KneePitch” motor and is dedicated to the tilt of the robot’s base. The final category is composed of three motors, “WheelFL”, “WheelFL”, and “WheelB”, which are responsible for the movement of the wheels for the robot’s displacement.

Formulas (1) and (3) are employed to ascertain the reliability calculation of the translation subsystem, with the values of each component of the system serving as the basis for this calculation. The following formula, Formula (10), is a detailed explanation of the manner in which this is done.

(10) $$R\left(t\right)=1-\left(1-PT1\right)\left(1-MOTOR\;KP\right)\left(1-PT2\right)$$

The final form of the translational intensity calculation is provided in Formula (11).

(11) $$\lambda_{translation}=-\frac{\ln\left[1-\left(1-e^{-\lambda_{pt1}t}\right)\left(1-e^{-\lambda_{MOTORKP}t}\right)\left(1-e^{-\lambda_{pt2}t}\right)\right]}{t}$$

#### 5.2.4. RBD for Safety

The final component, designated “Safety”, is evaluated in conjunction with the aforementioned three components. The three components are as follows: recognition, manipulation, and translation. This component’s objective is to prevent the occurrence of unforeseen incidents that may have a negative impact on the user, the environment, or even the robot itself. The subsystem is divided into four categories according to its components, with a total of 13 sensors. The first category consists of two infrared sensors, the second category consists of two sonar sensors, the third category consists of six laser-type sensors, and the fourth category consists of three laser actuators.

**Figure 13:**
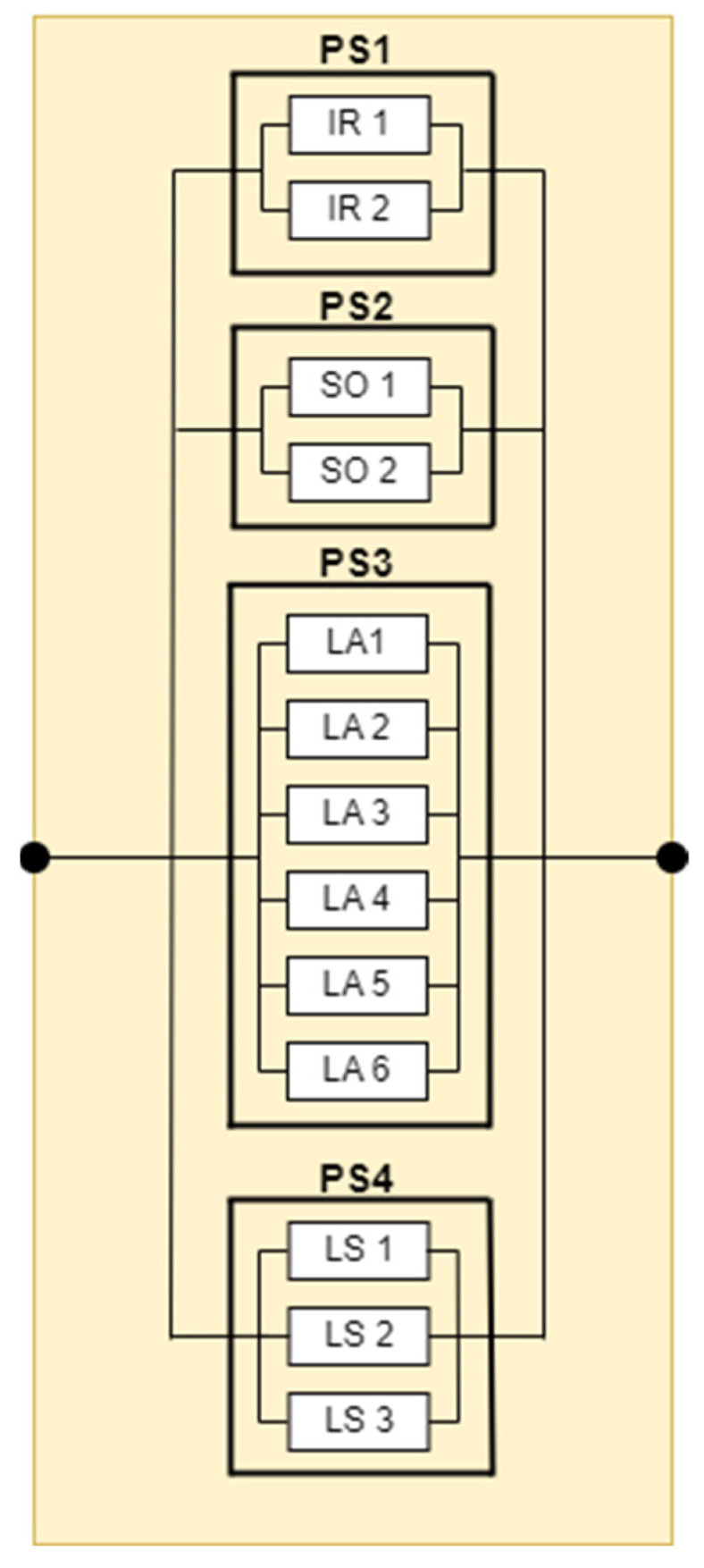
RBD diagram for safety.

The reliability of the safety subsystem, as illustrated in Figure 13, is calculated using Formula (12):

(12) $$R\left(t\right)=1-\left(1-PS1\right)\left(1-PS2\right)\left(1-PS3\right)\left(1-PS4\right)$$

In order to calculate the failure rate, the calculation is performed on each parallel connection, resulting in Formula (13), which takes the following form:

(13) $$\lambda_{safey}=-\frac{\ln\left[1-\left(1-e^{-\lambda_{ps1}t}\right)\left(1-e^{-\lambda_{ps2}t}\right)\left(1-e^{-\lambda_{ps3}t}\right)\left(1-e^{-\lambda_{ps4}t}\right)\right]}{t}$$

Table 2 lists the main observed failure rates.

To assess the system reliability effectively, we classified it into four parts: safety, recognition, translation, and handling. An individual MTBF analysis was performed for each component, depending on the proposed environment for the robot (see Table 3). The following formulas were used to calculate the failure intensity of each component listed in the table: Recognition Formula (7), Manipulation Formula (9), Transmission Formula (11), and Safety Formula (13). The MTBF calculation of each component is realized by using Formula (3).

In the field of reliability calculation for humanoid robots, the available literature is comparatively limited, resulting in a narrower scope of information compared to other fields that benefit from a larger volume of experiments.

Once the values for each component of the system have been established, the series components can be calculated according to Formula (14).

(14) $${R(t)}_{s\_total}={R(T)}_{recognition}\times {R(t)}_{manipulation}\times {R(t)}_{translation}\;{R(t)}_{s\_total}=0.95\times 0.99\times 0.99\;{R(t)}_{s\_total}=0.9993$$

The total reliability of the system can finally be determined by calculating in parallel with the safety component, as outlined in Formula (15).

(15) $${R(t)}_{total}=1-\left(1-{R(t)}_{s\_total}\right)\left(1-{R(t)}_{safety}\right)\;{R(t)}_{total}=1-\left(0.0007\right)\left(0.01\right)\;{R(t)}_{s\_total}=0.99993$$

To assess the reliability of the system, the PTC Windchill Quality Solution prediction method was used. The entire system has a reliability value of 0.999993, calculated by considering all the characteristics. Comparing our results with those found in the literature, it can be concluded that the reliability calculation performed here is highly efficient compared to previous examples.

In accordance with the methodology delineated in the preceding section, it is feasible to ascertain the reliability of any interacting robot. This sandbox incorporates a discernible sequence, commencing with the sectioning and configuration of the serial and parallel system components in the RBD diagram and culminating in the application of the provided formulas for the final system calculation.

#### 5.2.5. Fault Tree Analysis

A Fault Tree Analysis (FTA) is a graphical method of analyzing the causes of undesirable outcomes and faults within a system. The method involves traversing a tree from the top to the bottom, beginning at a single point at the apex and subsequently branching out at the base to encompass the entirety of the system’s states. The FTA employs the use of logic symbols to identify the underlying faults within the tree by analyzing the fundamental events. By plotting such a diagram on a system, it is possible to identify the fault chain by employing logic gates and events. By traversing the diagram, the veracity of events is established, and connections are made in order to identify the root cause of the failure [59].

**Figure 14:**
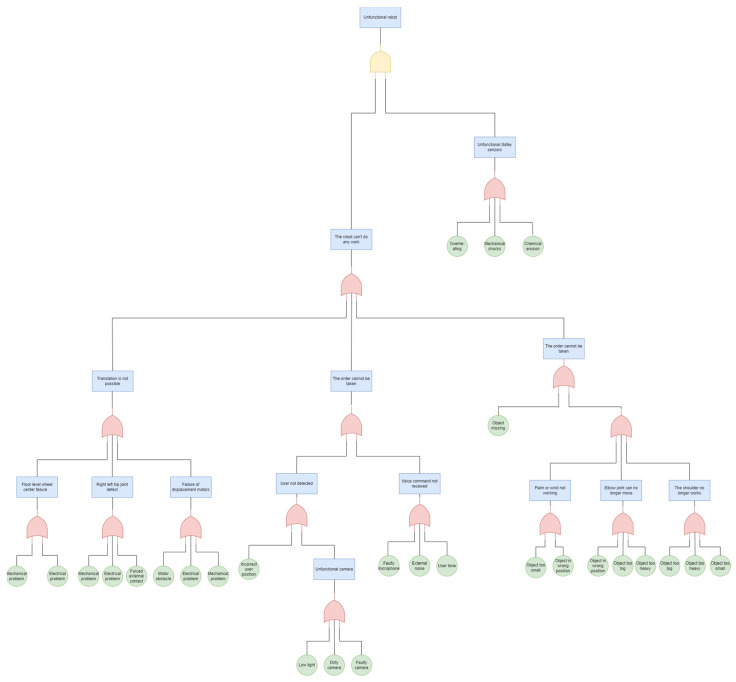
Fault tree diagram.

The FTA diagram of the studied system is designed in a way that allows for the analysis of any fault that may occur during a disruption in the system’s operation [60]. The diagram’s tree structure comprises five layers, enabling the identification of the system’s operational mode through the classification of operational classes, as illustrated in Figure 14. It is possible to identify certain faults at the second level of the tree, but the majority of the causes that could potentially lead to a system disturbance are present at the last level. By traversing the tree paths using Boolean logic, each system state that may potentially impede the smooth functioning and operation of the system is identified and evaluated.

#### 5.2.6. Markov System

In order to observe the progression of events and states within the system developed in this research, a Markov chain was utilized to track the probability of the system’s subsequent state over the course of one year. A Markov chain is a mathematical system based on probabilistic rules that undergoes transitions from one state to another. Its defining characteristic is that the transition to the future state is dependent only on the present state. This stochastic process exhibits memoryless characteristics. In the context of probability distributions, “memoryless” refers to a specific probability [61]. The Markov chain approach is a valuable tool in reliability studies due to its ability to model the various states that system components may undergo, including working, failure, and partial failure. These systems are represented by transitions between discrete states, wherein the probability of a system transitioning to a new state is contingent upon its current state and is independent of other states. In the field of reliability, the Markov chain is employed for modelling the components of a system over time. In order to model the Markov chain of this system, the following notations were employed: λ to express the failure intensity and μ to symbolize the average repair time.

**Figure 15:**
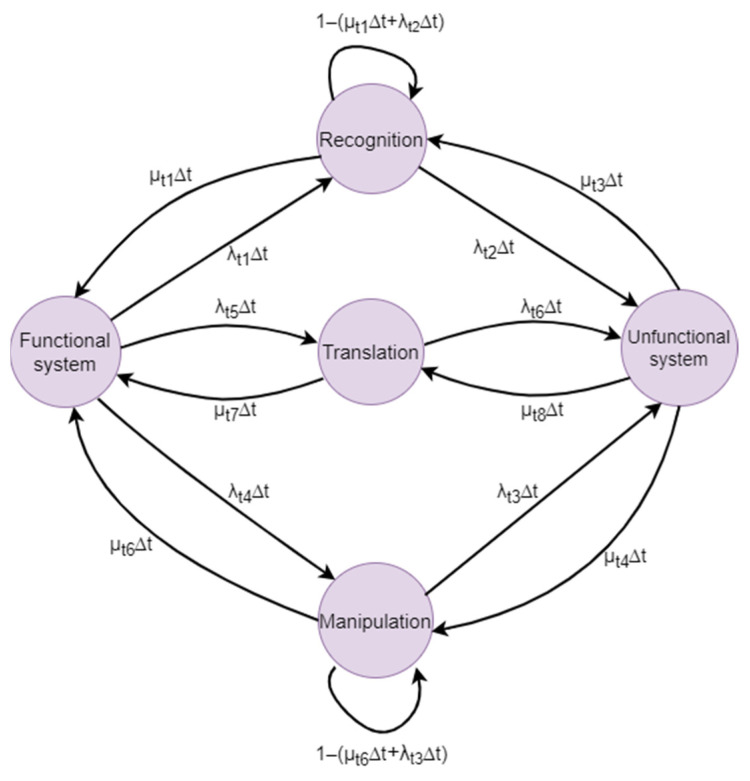
Minimized Markov chain of the system.

The diagram that exemplifies the structure of the system under investigation in this research is composed of five states: functional system, recognition, translation, manipulation, and non-functional system. The interconnectivity between these functional states is facilitated by twelve transitions, which illustrate the probability of a system transitioning from one state to another. A condensed representation of the Markov chain for the entire system is illustrated in Figure 15.

**Figure 16:**
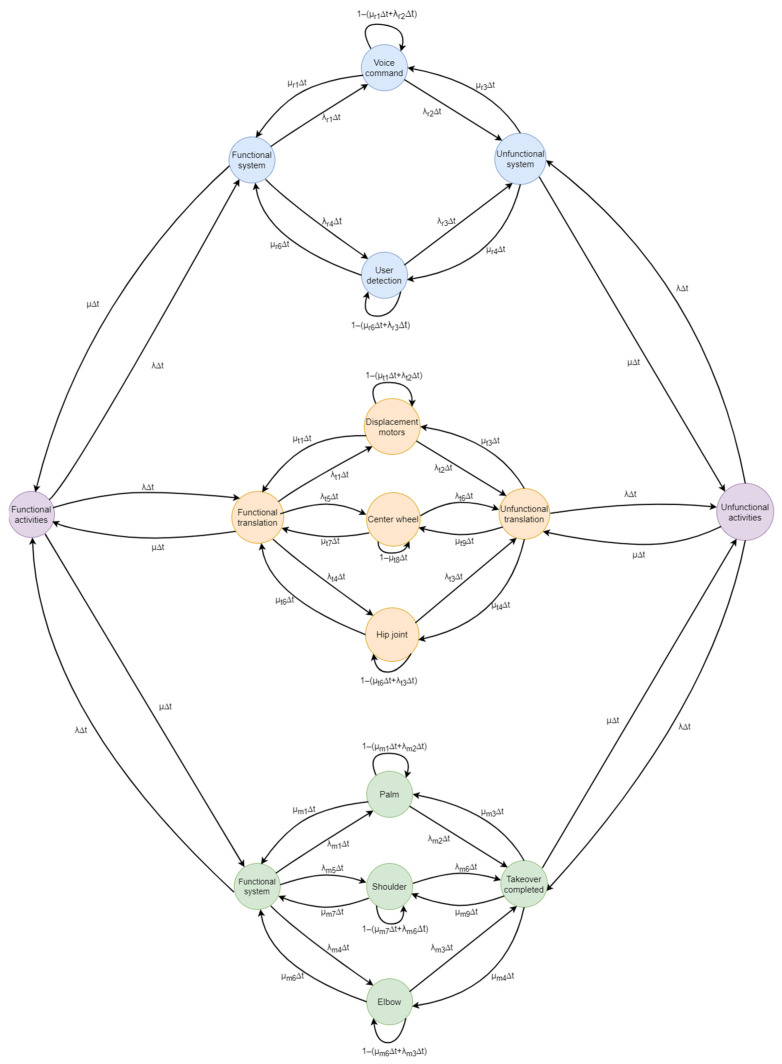
Markov chain of the system.

For a comprehensive illustration of the various states within the system, Figure 16 serves as a representative example. The diagram comprises sixteen states and forty-four transitions, which collectively express the probabilities of the entire system transitioning from one state to another.

In order to perform a comprehensive calculation of the system, it is necessary to construct an independent structure for each component involved. A case-specific Markov network is constructed based on the resulting subsystems, enabling the computation of the probability of the next state for each of the three cases. The three processes are those of recognition, manipulation, and translation.

#### 5.2.7. Markov Recognition

**Figure 17:**
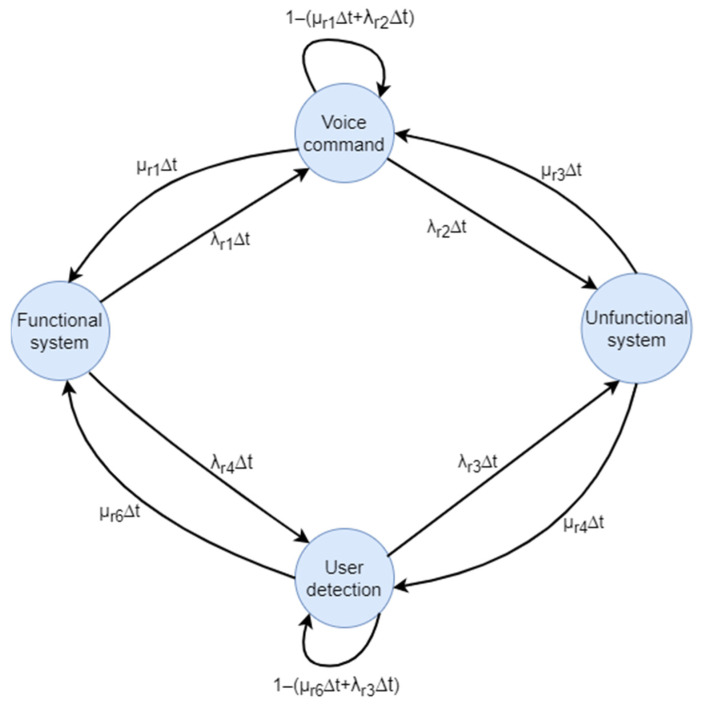
Markov chain of the recognition system.

The initial component, designated “Recognition”, encompasses ten transitions and four states: system functional, voice command, user detection, and system not functional. This configuration is illustrated in Figure 17.

Once the Markov chain has been established, the transition matrix can be constructed, thereby enabling the observation of the system’s behavior over time as it progresses from one state to another. This is a method for visualizing all transition probabilities between the states of the system. In constructing the matrix, the number of states is taken into account, with the number of rows and columns equal to the number of states in the system. Each element of the matrix represents the probability of transition from one state to another state in the system. When the matrix is visualized using Formula (16), the element in column position two, row one represents the transition from state one to state two, which is visible on the graph as λΔt.

In accordance with the realized Markov chain illustrated in Figure 17, the transition matrix pertinent to the recognition subsystem is formulated in accordance with the specifications set forth in Formula (16).

(16) $$\left[\begin{array}{cccc} 1-\lambda_{r1,4}\Delta t & \lambda_{r1}\Delta t & \lambda_{r4}\Delta t & 0\\ {µ}_{r1}∆t & 1-({µ}_{r1}∆t+\lambda_{r2}\Delta t) & 0 & \lambda_{r2}\Delta t\\ {µ}_{r6}∆t & 0 & 1-({µ}_{r6}∆t+\lambda_{r3}\Delta t) & \lambda_{r3}\Delta t\\ 0 & {µ}_{r3}∆t & {µ}_{r4}∆t & 1-{µ}_{r3,4}∆t \end{array}\right]$$

#### 5.2.8. Markov Translation

**Figure 18:**
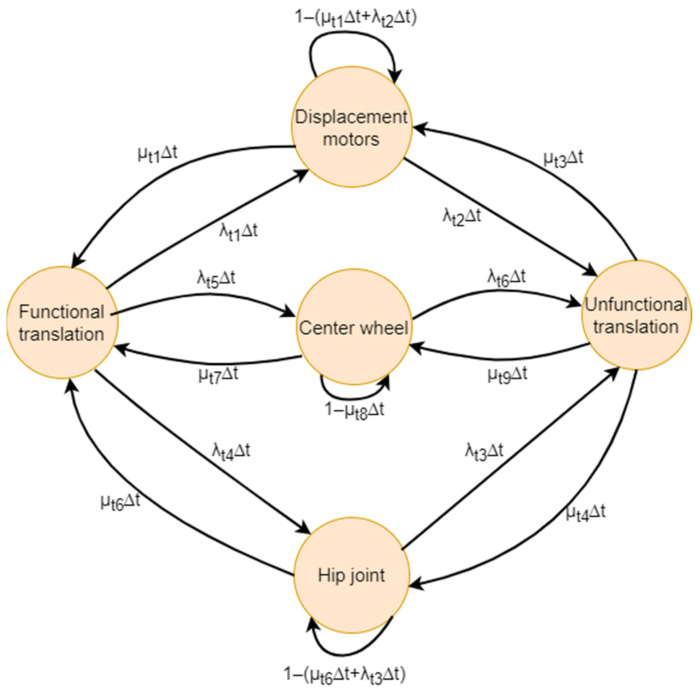
Markov chain of the translation system.

The Markov network specific to the second subsystem, designated as “translation”, is illustrated in Figure 18. The entire structure comprises twelve transitions and five states, namely the functional system, displacement motors, center wheel, hip joint, and non-functional translation.

The transition matrix, derived from the Markov chain of the translation subsystem, is presented in Formula (17) below:

(17) $$\left[\begin{array}{ccccc} 1-\lambda_{r1,4,5}\Delta t & \lambda_{t1}\Delta t & \lambda_{t5}\Delta t & \lambda_{t4}\Delta t & 0\\ {µ}_{t1}∆t & 1-({µ}_{t1}∆t+\lambda_{t2}\Delta t) & 0 & 0 & \lambda_{t2}\Delta t\\ {µ}_{t7}∆t & 0 & 1-({µ}_{t7}∆t+\lambda_{t6}\Delta t) & 0 & \lambda_{t6}\Delta t\\ {µ}_{t6}∆t & 0 & 0 & 1-({µ}_{t6}∆t+\lambda_{t3}\Delta t) & \lambda_{t3}\Delta t\\ 0 & {µ}_{t3}∆t & {µ}_{t9}∆t & {µ}_{t4}∆t & 1-{µ}_{t3,9,4}∆t \end{array}\right]$$

#### 5.2.9. Markov Manipulation

**Figure 19:**
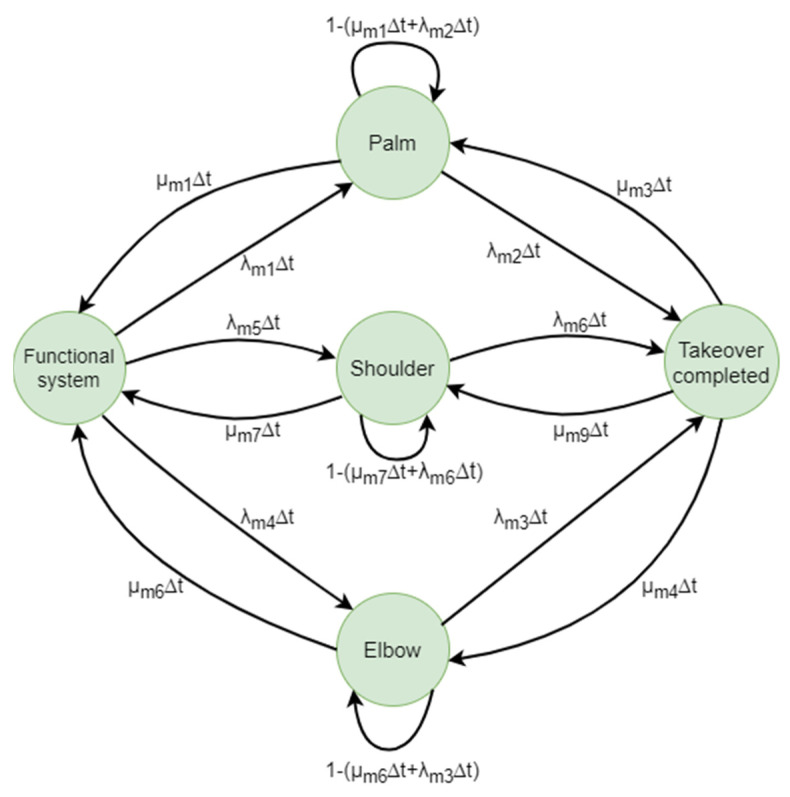
Markov chain of the manipulation system.

As illustrated in Figure 19, the Markov chain of the manipulation subsystem comprises twelve transitions and five states: functional system, palm, shoulder, elbow, and non-functional grasp.

The transition matrix of the manipulation subsystem is given by Formula (18):

(18) $$\left[\begin{array}{ccccc} 1-\lambda_{m1,4,5}\Delta t & \lambda_{m1}\Delta t & \lambda_{m5}\Delta t & \lambda_{m4}\Delta t & 0\\ {µ}_{m1}∆t & 1-(\lambda_{m2}∆t+\lambda {µ}_{m1}\Delta t) & 0 & 0 & \lambda_{m2}\Delta t\\ {µ}_{m7}∆t & 0 & 1-({µ}_{m7}∆t+\lambda_{m6}\Delta t) & 0 & \lambda_{m6}\Delta t\\ {µ}_{m6}∆t & 0 & 0 & 1-({µ}_{m6}∆t+\lambda_{m3}\Delta t) & \lambda_{m3}\Delta t\\ 0 & {µ}_{m3}∆t & {µ}_{m9}∆t & {µ}_{m4}∆t & 1-{µ}_{m3,9,4}∆t \end{array}\right]$$

The main benefit of this research is the presentation of the steps in which the Pepper robot reliability calculation is realized. These steps can be applied to other humanoid robot systems.

## 6. Discussion

The application was developed to improve office efficiency and cooperation among colleagues. Using a robot can bring several benefits to a company, including increased workload capacity, interaction between colleagues, and improved efficiency, which can positively impact the company’s budget.

Using an assistive robot in an office can significantly improve work efficiency and accuracy. Additionally, the appearance of such robots can improve interactions and boost morale, particularly for users who do not frequently interact with colleagues. To improve this development, there are plans to improve sound quality filtering and eliminate external stimuli. This will prevent the robot from interpreting any sound as a command, which currently causes the system to stall.

By calculating the reliability of the entire interaction system, it is possible to make further improvements to the overall cooperation process. This will result in a more efficient and streamlined process, which will be carried out without the need for human intervention and with a reduced number of errors. Thus, the incorporation of supplementary modules, such as a set of microphones that could substitute for existing microphones in the event of their malfunction, would inherently enhance the dependability of the command recognition system, subsequently improving the overall reliability of the system.

One significant limitation of the Pepper robot in such applications is its inability to traverse differences in floor levels. This hinders the robot’s activity, as it is unable to ascend to a higher floor level using a step. This aspect restricts its activities to those that can be conducted on a single level, and collaboration with colleagues on different levels can only be achieved by utilizing a ramp or elevator to traverse between floors. Furthermore, another limitation raises questions about the scope of applications in settings where the robot is required to exert significant force in the transportation of certain objects, given its constraints in gripping and holding an object exceeding 300 g with a single hand.

To strengthen the application, it is desirable to integrate and communicate with other humanoid robots. This will streamline the execution of processes and increase the reliability of the service. Integrating this new element will improve work accuracy by reducing human error rates. Additionally, it promotes cooperation and can enhance the acceptance of robots in social environments by increasing confidence in their use.

## 7. Conclusions

This paper presents a reliability analysis of a case study involving the realization of an interaction with the humanoid robot Pepper. The initial phase of the project entailed the creation of a software application designed to facilitate the work of the humanoid robot within an office setting. The objective was to enhance the efficiency of document handling and storage operations, while also maintaining the collective attention of employees. Once the application had been developed, the subsequent step was to analyze the reliability of the entire operating system. The initial step was to divide the whole structure into several independent modules, thus enabling the calculation of the modular reliability for the following components: recognition, translation, manipulation, and safety. This was achieved by creating a main RBD diagram. 

The second step was to create individual RBD diagrams for each module to be implemented, followed by the step of populating each module with the corresponding components that go into its formation, of which there were a total of 31. The creation of the components and the subsequent analysis of their data commenced with the structuring of the components according to the system in series and parallel. Once the system had a clear structure, the calculation phase was initiated with the objective of obtaining reliability. It was necessary to calculate each component and therefore, the failure intensity was determined according to Formula (3) by using the MTBF value of each subcomponent in the system. Once the intensity value was known, it was possible to calculate the reliability value of each component using Formula (1). These data were then used to carry out the parallel series calculation in order to find the reliability of each of the four main components of the system. Consequently, the final calculation was performed using the aforementioned methodology, resulting in a reliability value of 0.999993.

This method of assessing the reliability of a system can be applied to any system, thereby facilitating its utilization in a rapid and effective manner. Furthermore, the implementation of this approach can yield additional benefits, such as the generation of an accurate maintenance schedule that does not compromise the system’s optimal functioning or the safety of its users.

From the perspective of scalability, this manuscript can be utilized in a multitude of applications. By implementing the aforementioned steps, a comprehensive analysis of the reliability of a system can be conducted. The aforementioned analysis allows for a reduction in maintenance and operational costs, the implementation of appropriate maintenance through the use of MTBF evaluation, a long-term performance evaluation through Markov chain analysis, and a reduction in downtime through the minimization of failure intensity. As potential avenues for future development, it is feasible to initiate studies that would facilitate the implementation of this template on a system-by-system basis. Such studies could incorporate user interfaces to enhance the overall intuitiveness of the process, and they might also integrate a database comprising a substantial number of components that can be directly introduced with the specifications provided by the manufacturer.

## Figures and Tables

**Table 1:** Service robot trends.

Field	Commercial Name	Application
Healthcare	Aldebaran NAO robot (Aldebaran Robotics, Paris, France)	Physical exercise programs
	Care-o-Bot, hobbita (Fraunhofer IPA, Stuttgart, Germany)	Implement routines such as taking medicine
Home service	R1(Istituto Italiano di Tecnologia (IIT), Genova, Italy), Grillbot (Grillbot LLC, New York, NY, USA), Sony aibo (Sony Corporation, Tokyo, Japan)	Help in house tasks
Multi-purpose indoor environments	Festo Robotino (Festo, Esslingen am Neckar, Germany)	Indoor positioning system for service
	TurtleBot (Willow Garage, Palo Alto, CA, USA)	Wall detection and obstacle avoidance
	Beta-G (Gamma2 Robotics, Denver, CO, USA), Richtech (Richtech Robotics, Las Vegas, NV, USA)	Identify tables in a restaurant

**Table 2:** Top 10 component failure rates.

System	System Item	MTBF	Failure Rate
Recognition	Camera 1	50,000 h	0.0002 failures/h
	Camera 2	50,000 h	0.0002 failures/h
	Microphone 1	42,710 h	0.000023 failures/h
	Microphone 2	42,710 h	0.000023 failures/h
	Microphone 3	42,710 h	0.000023 failures/h
	Microphone 4	42,710 h	0.000023 failures/h
	Motor Yaw	100,000 h	0.00001 failures/h
	Motor Pitch	100,000 h	0.00001 failures/h
	RGBD LED	50,000 h	0.0002 failures/h
Manipulation	Motor Shoulder 1	100,000 h	0.00001 failures/h
	Motor Shoulder 2	100,000 h	0.00001 failures/h
	Motor Elbow 1	100,000 h	0.00001 failures/h
	Motor Elbow 2	100,000 h	0.00001 failures/h
	Motor Joint 1	100,000 h	0.00001 failures/h
	Motor Joint 2	100,000 h	0.00001 failures/h
Translation	Motor HipRoll	70,000 h	0.00001429 failures/h
	Motor HipPitch	70,000 h	0.00001429 failures/h
	Motor_KneePitch	70,000 h	0.00001429 failures/h
	Motor_WheelB 1	70,000 h	0.00001429 failures/h
	Motor_WheelB 2	70,000 h	0.00001429 failures/h
	Motor_WheelB 3	70,000 h	0.00001429 failures/h
Safety	Laser Sensor 1	40,000 h	0.000025 failures/h
	Laser Sensor 2	40,000 h	0.000025 failures/h
	Laser Sensor 3	40,000 h	0.000025 failures/h
	Infra Red 1	35,000 h	0.00002857 failures/h
	Infra Red 2	35,000 h	0.00002857 failures/h
	Laser Actuators 1	20,000 h	0.0005 failures/h
	Laser Actuators 2	20,000 h	0.0005 failures/h
	Laser Actuators 3	20,000 h	0.0005 failures/h
	Laser Actuators 4	20,000 h	0.0005 failures/h
	Laser Actuators 5	20,000 h	0.0005 failures/h
	Laser Actuators 6	20,000 h	0.0005 failures/h
	Sonars 1	200,000 h	0.000005 failures/h
	Sonars 2	200,000 h	0.000005 failures/h

**Table 3:** The value of MTBF and the failure rate for each component.

Module	MTBF	Failure Rate
Recognition	9670 h	0.00010341 failures/h
Manipulation	1667 h	0.00006 failures/h
Safety	22.62 h	0.00044209 failures/h
Translation	11,667 h	0.00008571 failures/h

## Data Availability

The datasets presented in the paper are not readily available because the data are part of an ongoing study. Requests to access the datasets should be directed to the authors.
